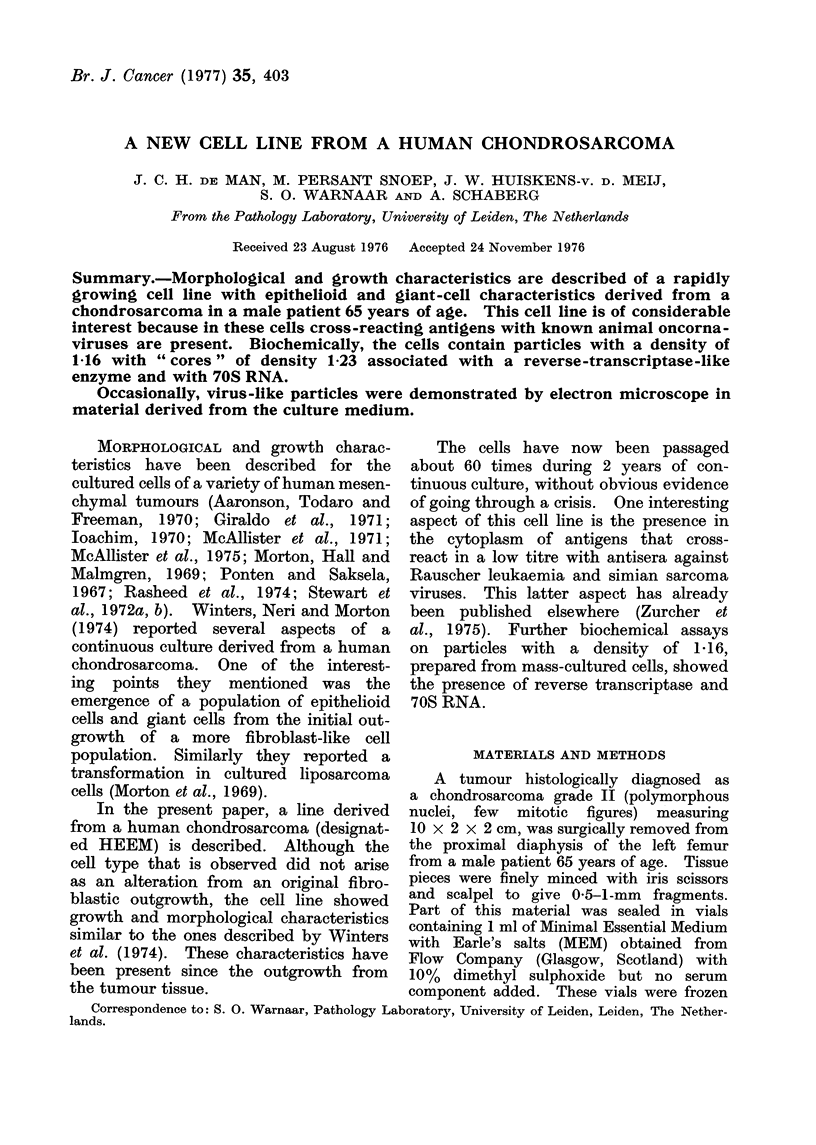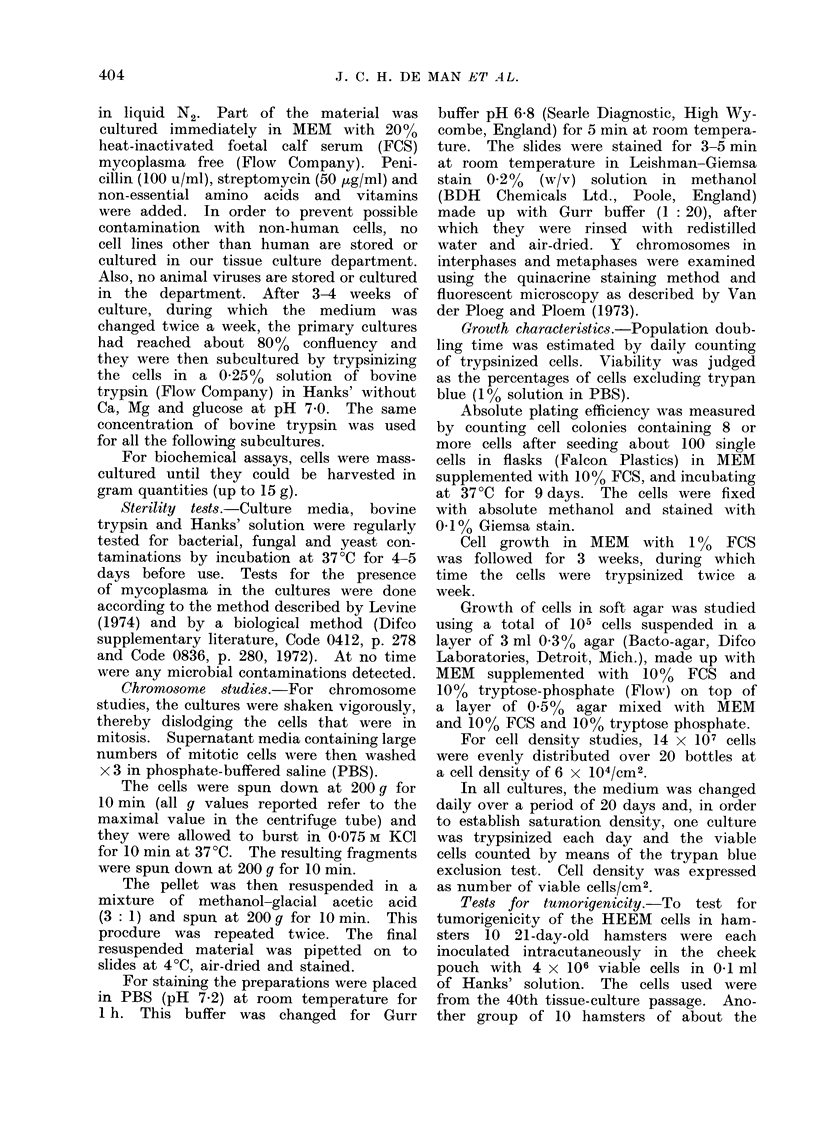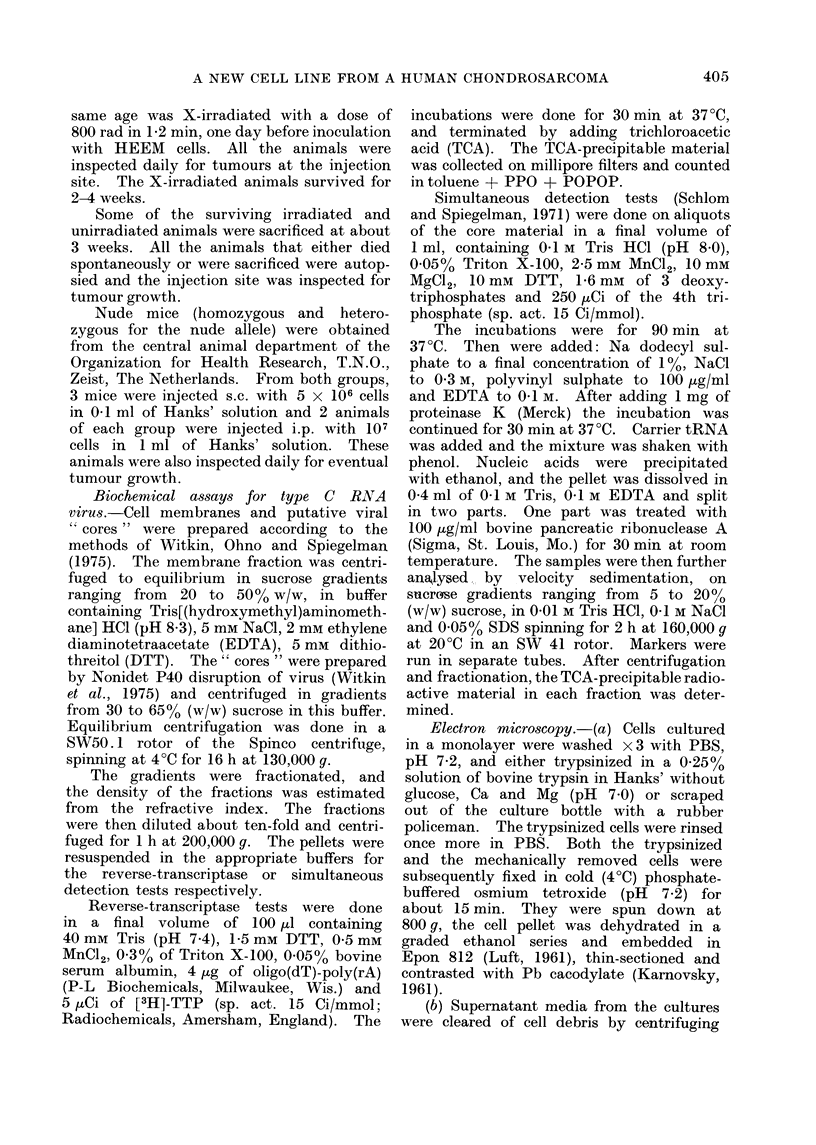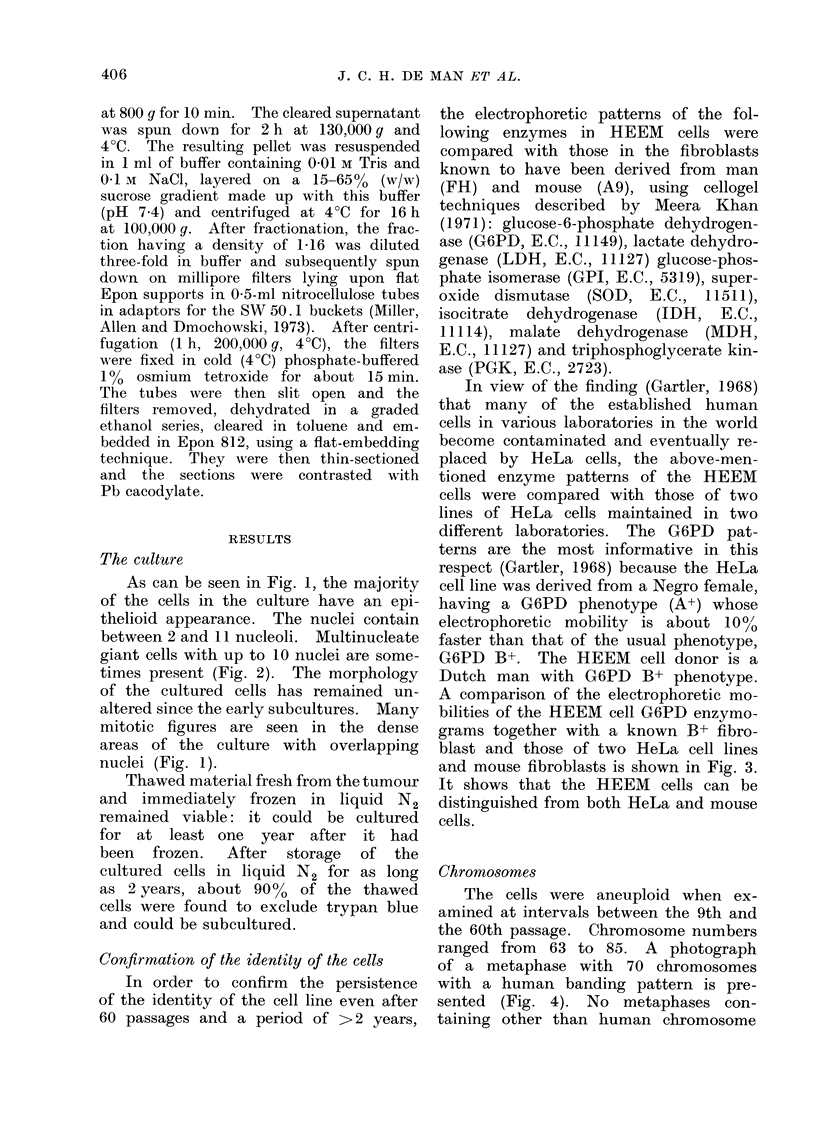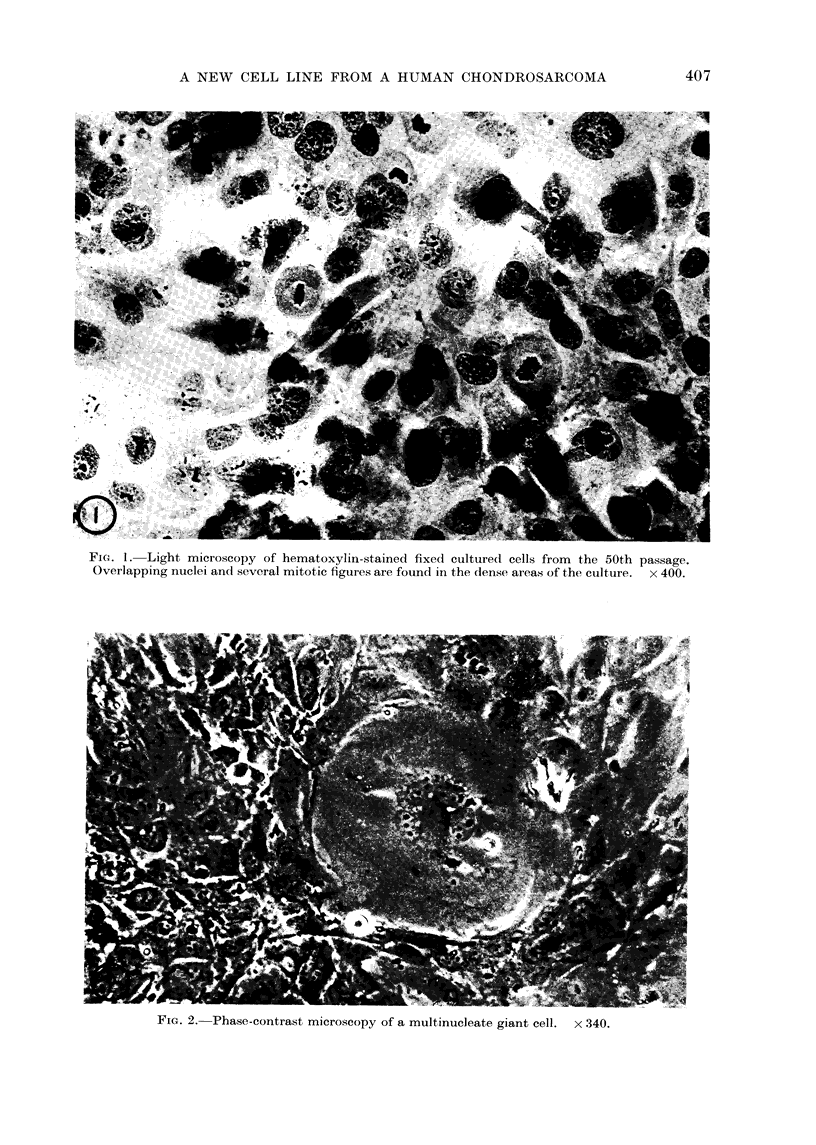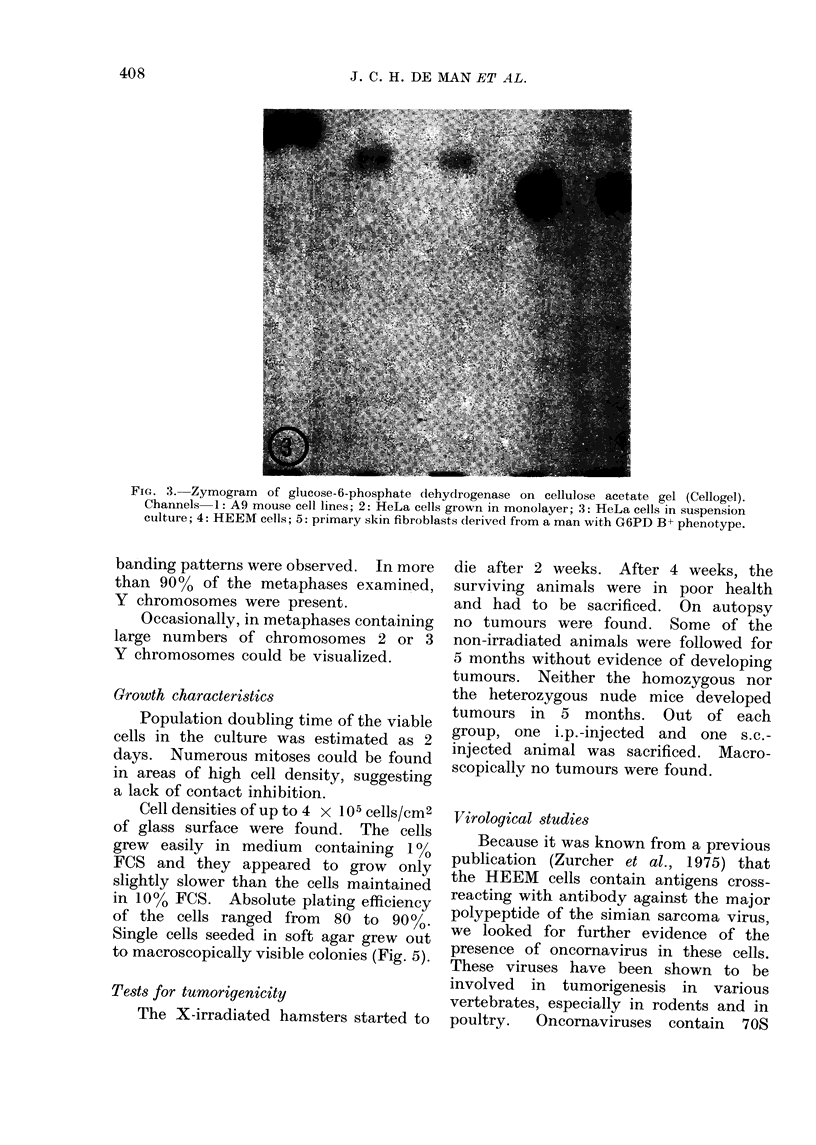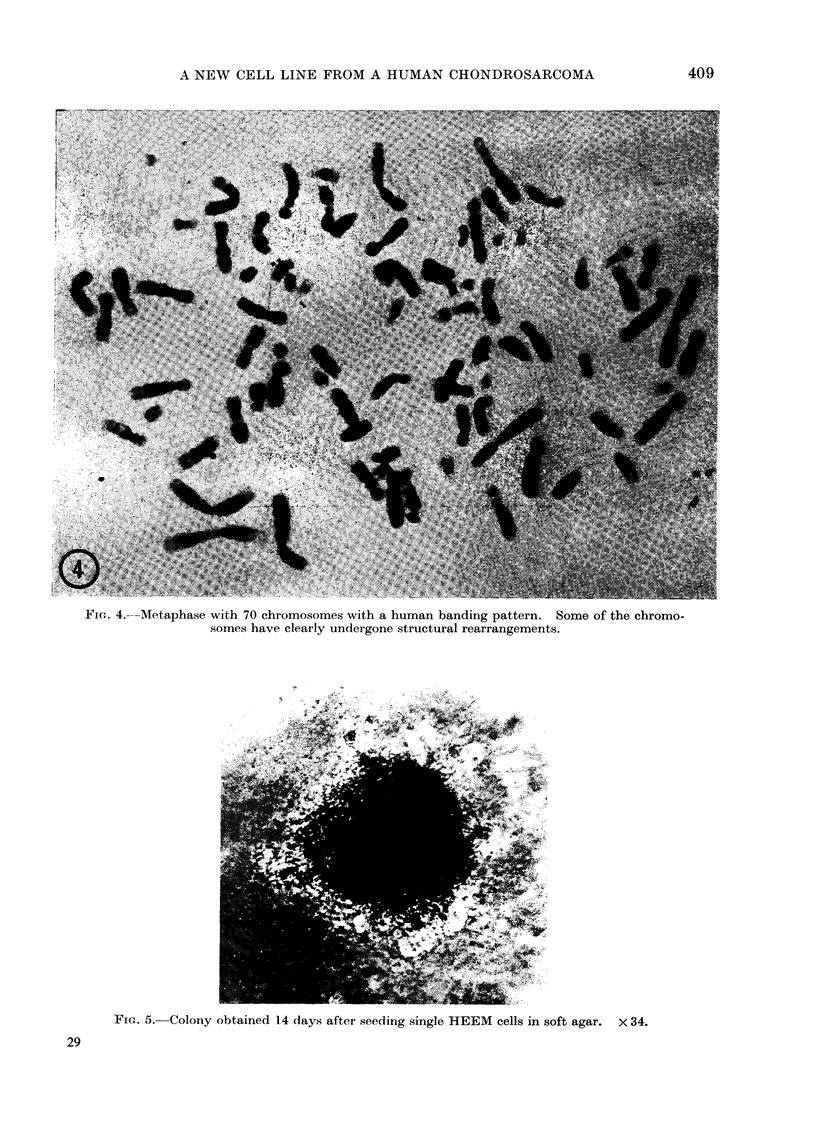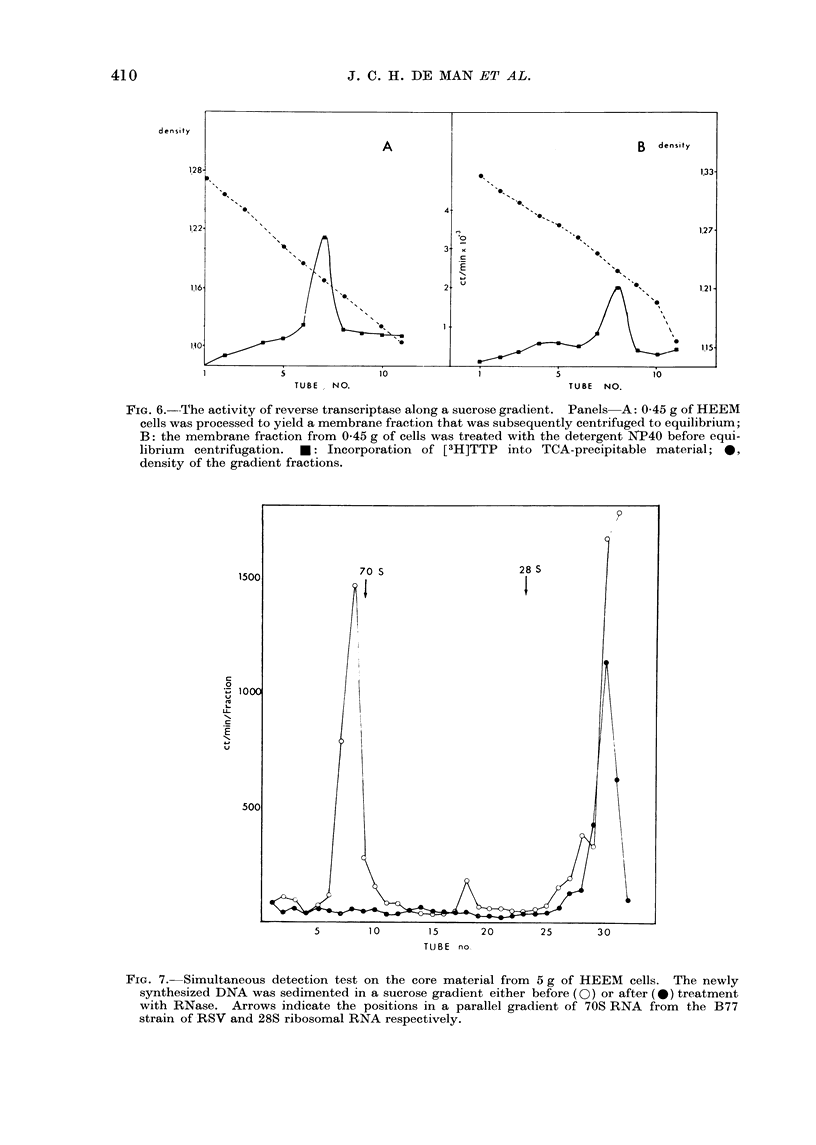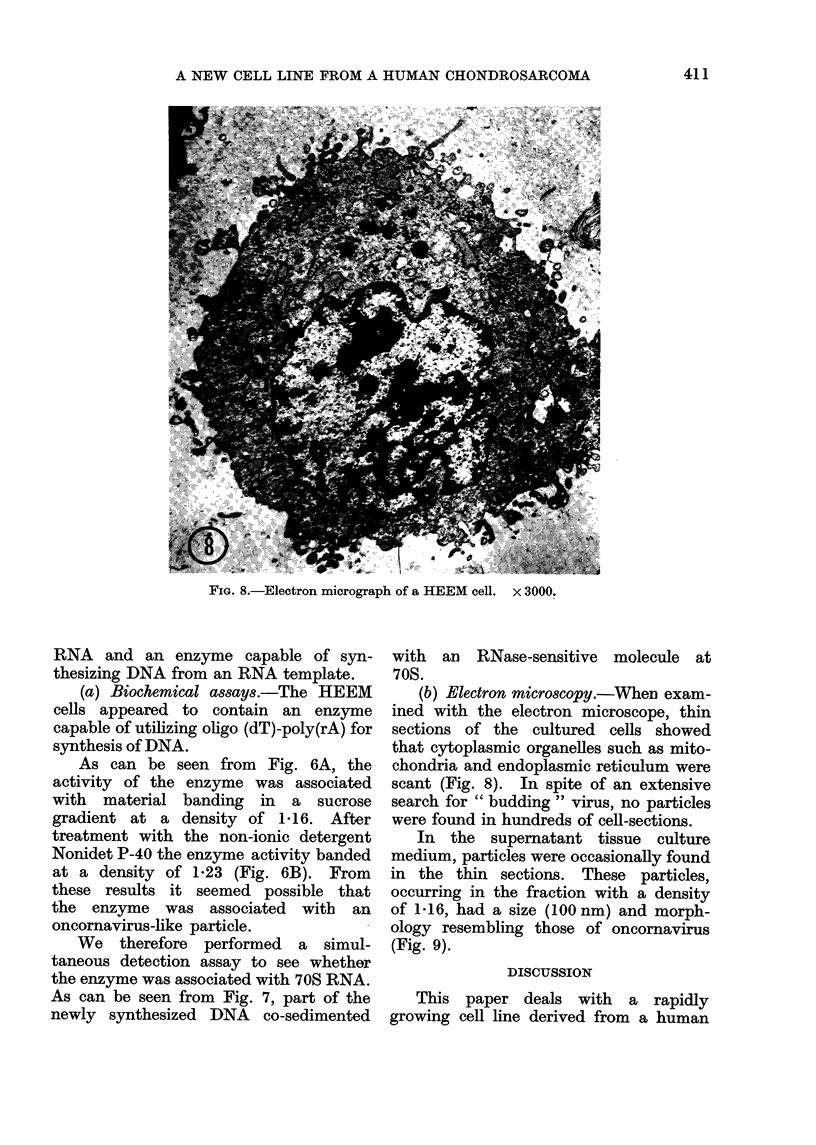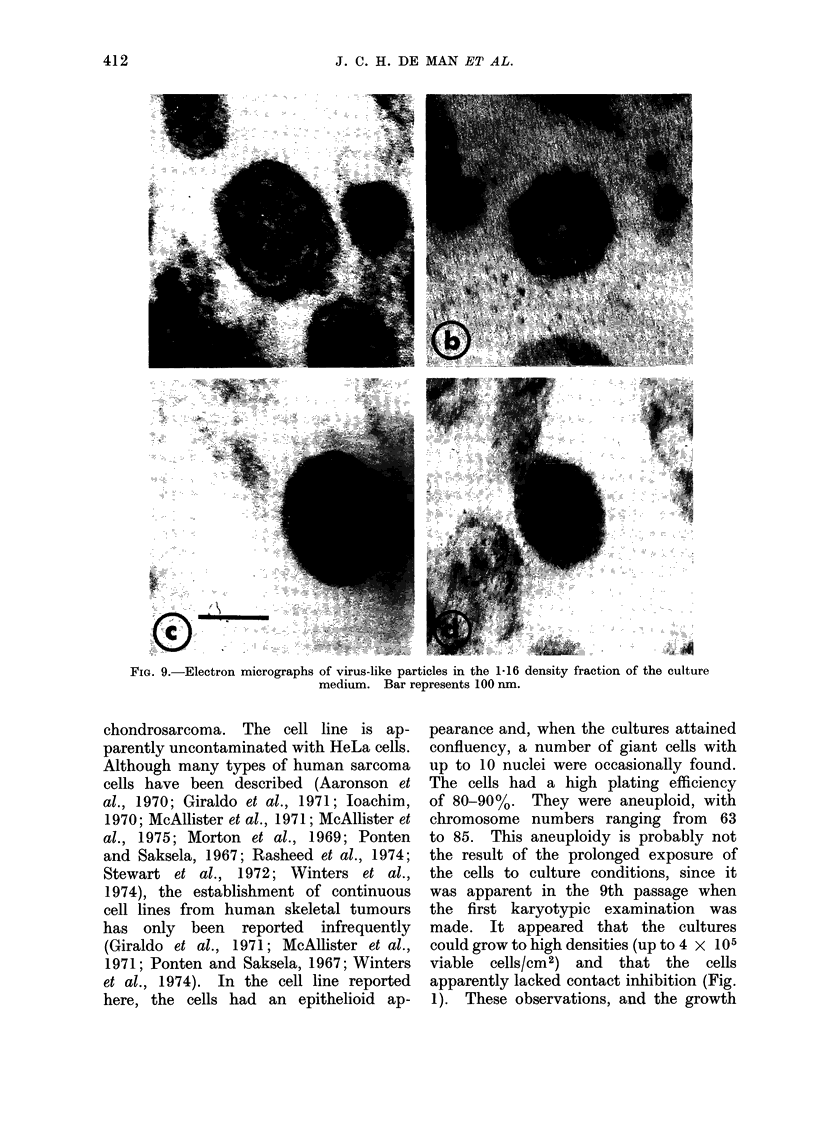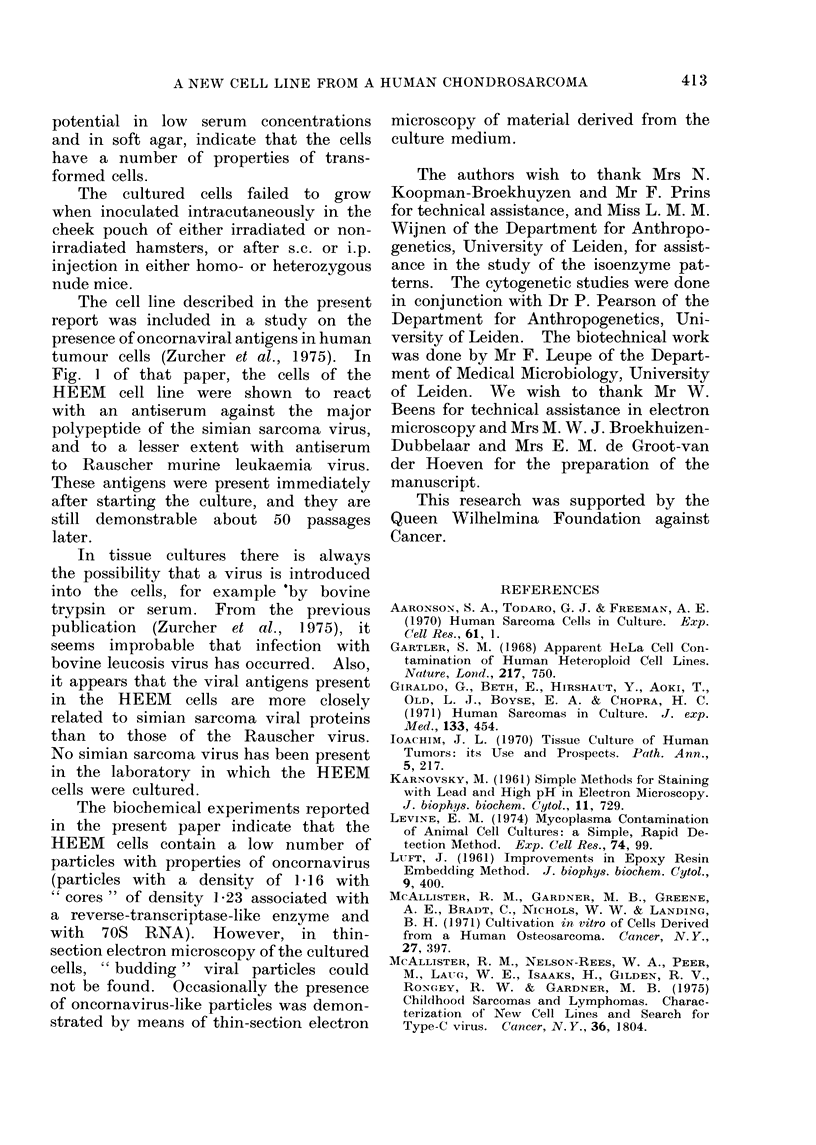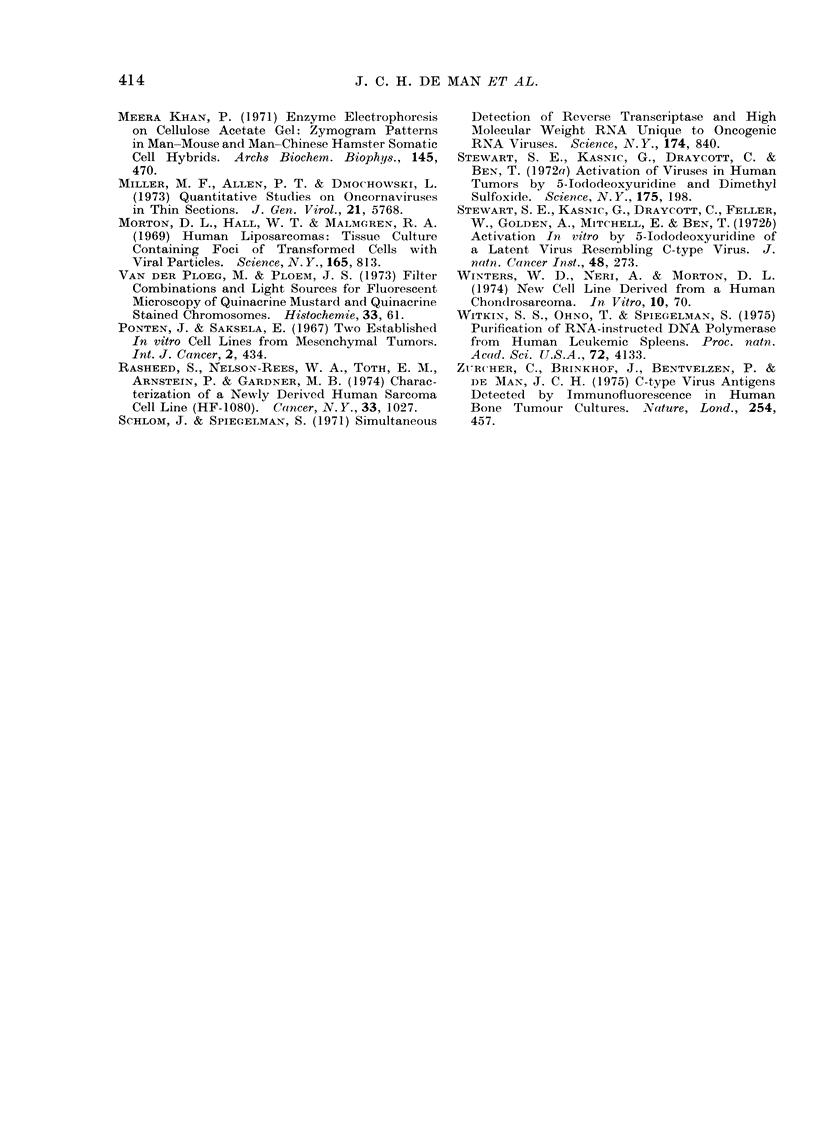# A new cell line from a human chondrosarcoma.

**DOI:** 10.1038/bjc.1977.61

**Published:** 1977-04

**Authors:** J. C. de Man, M. P. Snoep, J. W. Huiskens-van der Meij, S. O. Warnaar, A. Schaberg

## Abstract

**Images:**


*Br. J. Cancer* (1977) **35**, 403

### A NEW CELL LINE FROM A HUMAN CHONDROSARCOMA

J. C. H. DE MAN, M. PERSANT SNOEP, J. W. HUISKENS-V. D. MEIJ,
S. O. WARNAAR AND A. SCHABERG

*From the Pathology Laboratory, University of Leiden, The Netherlands*

Received 23 August 1976 Accepted 24 November 1976

**Summary.—Morphological and growth characteristics are described of a rapidly growing cell line with epithelioid and giant-cell characteristics derived from a chondrosarcoma in a male patient 65 years of age. This cell line is of considerable interest because in these cells cross-reacting antigens with known animal oncornaviruses are present. Biochemically, the cells contain particles with a density of 1·16 with " cores " of density 1·23 associated with a reverse-transcriptase-like enzyme and with 70S RNA.**

**Occasionally, virus-like particles were demonstrated by electron microscope in material derived from the culture medium.**

MORPHOLOGICAL and growth characteristics have been described for the cultured cells of a variety of human mesenchymal tumours (Aaronson, Todaro and Freeman, 1970; Giraldo *et al.*, 1971; Ioachim, 1970; McAllister *et al.*, 1971; McAllister *et al.*, 1975; Morton, Hall and Malmgren, 1969; Ponten and Saksela, 1967; Rasheed *et al.*, 1974; Stewart *et al.*, 1972*a*, *b*). Winters, Neri and Morton (1974) reported several aspects of a continuous culture derived from a human chondrosarcoma. One of the interesting points they mentioned was the emergence of a population of epithelioid cells and giant cells from the initial outgrowth of a more fibroblast-like cell population. Similarly they reported a transformation in cultured liposarcoma cells (Morton *et al.*, 1969).

In the present paper, a line derived from a human chondrosarcoma (designated HEEM) is described. Although the cell type that is observed did not arise as an alteration from an original fibroblastic outgrowth, the cell line showed growth and morphological characteristics similar to the ones described by Winters *et al.* (1974). These characteristics have been present since the outgrowth from the tumour tissue.

The cells have now been passaged about 60 times during 2 years of continuous culture, without obvious evidence of going through a crisis. One interesting aspect of this cell line is the presence in the cytoplasm of antigens that cross-react in a low titre with antisera against Rauscher leukaemia and simian sarcoma viruses. This latter aspect has already been published elsewhere (Zurcher *et al.*, 1975). Further biochemical assays on particles with a density of 1·16, prepared from mass-cultured cells, showed the presence of reverse transcriptase and 70S RNA.

### MATERIALS AND METHODS

A tumour histologically diagnosed as a chondrosarcoma grade II (polymorphous nuclei, few mitotic figures) measuring 10 × 2 × 2 cm, was surgically removed from the proximal diaphysis of the left femur from a male patient 65 years of age. Tissue pieces were finely minced with iris scissors and scalpel to give 0·5–1-mm fragments. Part of this material was sealed in vials containing 1 ml of Minimal Essential Medium with Earle's salts (MEM) obtained from Flow Company (Glasgow, Scotland) with 10% dimethyl sulphoxide but no serum component added. These vials were frozen

Correspondence to: S. O. Warnaar, Pathology Laboratory, University of Leiden, Leiden, The Netherlands.

in liquid $N_2$. Part of the material was cultured immediately in MEM with 20% heat-inactivated foetal calf serum (FCS) mycoplasma free (Flow Company). Penicillin (100 u/ml), streptomycin (50 $\mu$g/ml) and non-essential amino acids and vitamins were added. In order to prevent possible contamination with non-human cells, no cell lines other than human are stored or cultured in our tissue culture department. Also, no animal viruses are stored or cultured in the department. After 3–4 weeks of culture, during which the medium was changed twice a week, the primary cultures had reached about 80% confluency and they were then subcultured by trypsinizing the cells in a 0·25% solution of bovine trypsin (Flow Company) in Hanks' without Ca, Mg and glucose at pH 7·0. The same concentration of bovine trypsin was used for all the following subcultures.

For biochemical assays, cells were mass-cultured until they could be harvested in gram quantities (up to 15 g).

*Sterility tests.*—Culture media, bovine trypsin and Hanks' solution were regularly tested for bacterial, fungal and yeast contaminations by incubation at 37°C for 4–5 days before use. Tests for the presence of mycoplasma in the cultures were done according to the method described by Levine (1974) and by a biological method (Difco supplementary literature, Code 0412, p. 278 and Code 0836, p. 280, 1972). At no time were any microbial contaminations detected.

*Chromosome studies.*—For chromosome studies, the cultures were shaken vigorously, thereby dislodging the cells that were in mitosis. Supernatant media containing large numbers of mitotic cells were then washed ×3 in phosphate-buffered saline (PBS).

The cells were spun down at 200 *g* for 10 min (all *g* values reported refer to the maximal value in the centrifuge tube) and they were allowed to burst in 0·075 M KCl for 10 min at 37°C. The resulting fragments were spun down at 200 *g* for 10 min.

The pellet was then resuspended in a mixture of methanol–glacial acetic acid (3 : 1) and spun at 200 *g* for 10 min. This procdure was repeated twice. The final resuspended material was pipetted on to slides at 4°C, air-dried and stained.

For staining the preparations were placed in PBS (pH 7·2) at room temperature for 1 h. This buffer was changed for Gurr buffer pH 6·8 (Searle Diagnostic, High Wycombe, England) for 5 min at room temperature. The slides were stained for 3–5 min at room temperature in Leishman–Giemsa stain 0·2% (w/v) solution in methanol (BDH Chemicals Ltd., Poole, England) made up with Gurr buffer (1 : 20), after which they were rinsed with redistilled water and air-dried. Y chromosomes in interphases and metaphases were examined using the quinacrine staining method and fluorescent microscopy as described by Van der Ploeg and Ploem (1973).

*Growth characteristics.*—Population doubling time was estimated by daily counting of trypsinized cells. Viability was judged as the percentages of cells excluding trypan blue (1% solution in PBS).

Absolute plating efficiency was measured by counting cell colonies containing 8 or more cells after seeding about 100 single cells in flasks (Falcon Plastics) in MEM supplemented with 10% FCS, and incubating at 37°C for 9 days. The cells were fixed with absolute methanol and stained with 0·1% Giemsa stain.

Cell growth in MEM with 1% FCS was followed for 3 weeks, during which time the cells were trypsinized twice a week.

Growth of cells in soft agar was studied using a total of $10^5$ cells suspended in a layer of 3 ml 0·3% agar (Bacto-agar, Difco Laboratories, Detroit, Mich.), made up with MEM supplemented with 10% FCS and 10% tryptose-phosphate (Flow) on top of a layer of 0·5% agar mixed with MEM and 10% FCS and 10% tryptose phosphate.

For cell density studies, $14 \times 10^7$ cells were evenly distributed over 20 bottles at a cell density of $6 \times 10^4/cm^2$.

In all cultures, the medium was changed daily over a period of 20 days and, in order to establish saturation density, one culture was trypsinized each day and the viable cells counted by means of the trypan blue exclusion test. Cell density was expressed as number of viable cells/$cm^2$.

*Tests for tumorigenicity.*—To test for tumorigenicity of the HEEM cells in hamsters 10 21-day-old hamsters were each inoculated intracutaneously in the cheek pouch with $4 \times 10^6$ viable cells in 0·1 ml of Hanks' solution. The cells used were from the 40th tissue-culture passage. Another group of 10 hamsters of about the same age was X-irradiated with a dose of 800 rad in 1·2 min, one day before inoculation with HEEM cells. All the animals were inspected daily for tumours at the injection site. The X-irradiated animals survived for 2–4 weeks.

Some of the surviving irradiated and unirradiated animals were sacrificed at about 3 weeks. All the animals that either died spontaneously or were sacrificed were autopsied and the injection site was inspected for tumour growth.

Nude mice (homozygous and heterozygous for the nude allele) were obtained from the central animal department of the Organization for Health Research, T.N.O., Zeist, The Netherlands. From both groups, 3 mice were injected s.c. with $5 \times 10^6$ cells in 0·1 ml of Hanks' solution and 2 animals of each group were injected i.p. with $10^7$ cells in 1 ml of Hanks' solution. These animals were also inspected daily for eventual tumour growth.

*Biochemical assays for type C RNA virus.*—Cell membranes and putative viral "cores" were prepared according to the methods of Witkin, Ohno and Spiegelman (1975). The membrane fraction was centrifuged to equilibrium in sucrose gradients ranging from 20 to 50% w/w, in buffer containing Tris[(hydroxymethyl)aminomethane] HCl (pH 8·3), 5 mM NaCl, 2 mM ethylene diaminotetraacetate (EDTA), 5 mM dithiothreitol (DTT). The "cores" were prepared by Nonidet P40 disruption of virus (Witkin *et al.*, 1975) and centrifuged in gradients from 30 to 65% (w/w) sucrose in this buffer. Equilibrium centrifugation was done in a SW50.1 rotor of the Spinco centrifuge, spinning at 4°C for 16 h at 130,000 *g*.

The gradients were fractionated, and the density of the fractions was estimated from the refractive index. The fractions were then diluted about ten-fold and centrifuged for 1 h at 200,000 *g*. The pellets were resuspended in the appropriate buffers for the reverse-transcriptase or simultaneous detection tests respectively.

Reverse-transcriptase tests were done in a final volume of 100 μl containing 40 mM Tris (pH 7·4), 1·5 mM DTT, 0·5 mM $MnCl_2$, 0·3% of Triton X-100, 0·05% bovine serum albumin, 4 μg of oligo(dT)-poly(rA) (P-L Biochemicals, Milwaukee, Wis.) and 5 μCi of [$^3$H]-TTP (sp. act. 15 Ci/mmol; Radiochemicals, Amersham, England). The incubations were done for 30 min at 37°C, and terminated by adding trichloroacetic acid (TCA). The TCA-precipitable material was collected on millipore filters and counted in toluene + PPO + POPOP.

Simultaneous detection tests (Schlom and Spiegelman, 1971) were done on aliquots of the core material in a final volume of 1 ml, containing 0·1 M Tris HCl (pH 8·0), 0·05% Triton X-100, 2·5 mM $MnCl_2$, 10 mM $MgCl_2$, 10 mM DTT, 1·6 mM of 3 deoxytriphosphates and 250 μCi of the 4th triphosphate (sp. act. 15 Ci/mmol).

The incubations were for 90 min at 37°C. Then were added: Na dodecyl sulphate to a final concentration of 1%, NaCl to 0·3 M, polyvinyl sulphate to 100 μg/ml and EDTA to 0·1 M. After adding 1 mg of proteinase K (Merck) the incubation was continued for 30 min at 37°C. Carrier tRNA was added and the mixture was shaken with phenol. Nucleic acids were precipitated with ethanol, and the pellet was dissolved in 0·4 ml of 0·1 M Tris, 0·1 M EDTA and split in two parts. One part was treated with 100 μg/ml bovine pancreatic ribonuclease A (Sigma, St. Louis, Mo.) for 30 min at room temperature. The samples were then further analysed by velocity sedimentation, on sucrose gradients ranging from 5 to 20% (w/w) sucrose, in 0·01 M Tris HCl, 0·1 M NaCl and 0·05% SDS spinning for 2 h at 160,000 *g* at 20°C in an SW 41 rotor. Markers were run in separate tubes. After centrifugation and fractionation, the TCA-precipitable radioactive material in each fraction was determined.

*Electron microscopy.*—(*a*) Cells cultured in a monolayer were washed ×3 with PBS, pH 7·2, and either trypsinized in a 0·25% solution of bovine trypsin in Hanks' without glucose, Ca and Mg (pH 7·0) or scraped out of the culture bottle with a rubber policeman. The trypsinized cells were rinsed once more in PBS. Both the trypsinized and the mechanically removed cells were subsequently fixed in cold (4°C) phosphate-buffered osmium tetroxide (pH 7·2) for about 15 min. They were spun down at 800 *g*, the cell pellet was dehydrated in a graded ethanol series and embedded in Epon 812 (Luft, 1961), thin-sectioned and contrasted with Pb cacodylate (Karnovsky, 1961).

(*b*) Supernatant media from the cultures were cleared of cell debris by centrifuging at 800 *g* for 10 min. The cleared supernatant was spun down for 2 h at 130,000 *g* and 4°C. The resulting pellet was resuspended in 1 ml of buffer containing 0·01 M Tris and 0·1 M NaCl, layered on a 15–65% (w/w) sucrose gradient made up with this buffer (pH 7·4) and centrifuged at 4°C for 16 h at 100,000 *g*. After fractionation, the fraction having a density of 1·16 was diluted three-fold in buffer and subsequently spun down on millipore filters lying upon flat Epon supports in 0·5-ml nitrocellulose tubes in adaptors for the SW 50.1 buckets (Miller, Allen and Dmochowski, 1973). After centrifugation (1 h, 200,000 *g*, 4°C), the filters were fixed in cold (4°C) phosphate-buffered 1% osmium tetroxide for about 15 min. The tubes were then slit open and the filters removed, dehydrated in a graded ethanol series, cleared in toluene and embedded in Epon 812, using a flat-embedding technique. They were then thin-sectioned and the sections were contrasted with Pb cacodylate.

### RESULTS

### *The culture*

As can be seen in Fig. 1, the majority of the cells in the culture have an epithelioid appearance. The nuclei contain between 2 and 11 nucleoli. Multinucleate giant cells with up to 10 nuclei are sometimes present (Fig. 2). The morphology of the cultured cells has remained unaltered since the early subcultures. Many mitotic figures are seen in the dense areas of the culture with overlapping nuclei (Fig. 1).

Thawed material fresh from the tumour and immediately frozen in liquid $N_2$ remained viable: it could be cultured for at least one year after it had been frozen. After storage of the cultured cells in liquid $N_2$ for as long as 2 years, about 90% of the thawed cells were found to exclude trypan blue and could be subcultured.

### *Confirmation of the identity of the cells*

In order to confirm the persistence of the identity of the cell line even after 60 passages and a period of >2 years, the electrophoretic patterns of the following enzymes in HEEM cells were compared with those in the fibroblasts known to have been derived from man (FH) and mouse (A9), using cellogel techniques described by Meera Khan (1971): glucose-6-phosphate dehydrogenase (G6PD, E.C., 11149), lactate dehydrogenase (LDH, E.C., 11127) glucose-phosphate isomerase (GPI, E.C., 5319), superoxide dismutase (SOD, E.C., 11511), isocitrate dehydrogenase (IDH, E.C., 11114), malate dehydrogenase (MDH, E.C., 11127) and triphosphoglycerate kinase (PGK, E.C., 2723).

In view of the finding (Gartler, 1968) that many of the established human cells in various laboratories in the world become contaminated and eventually replaced by HeLa cells, the above-mentioned enzyme patterns of the HEEM cells were compared with those of two lines of HeLa cells maintained in two different laboratories. The G6PD patterns are the most informative in this respect (Gartler, 1968) because the HeLa cell line was derived from a Negro female, having a G6PD phenotype ($A^+$) whose electrophoretic mobility is about 10% faster than that of the usual phenotype, G6PD $B^+$. The HEEM cell donor is a Dutch man with G6PD $B^+$ phenotype. A comparison of the electrophoretic mobilities of the HEEM cell G6PD enzymograms together with a known $B^+$ fibroblast and those of two HeLa cell lines and mouse fibroblasts is shown in Fig. 3. It shows that the HEEM cells can be distinguished from both HeLa and mouse cells.

### *Chromosomes*

The cells were aneuploid when examined at intervals between the 9th and the 60th passage. Chromosome numbers ranged from 63 to 85. A photograph of a metaphase with 70 chromosomes with a human banding pattern is presented (Fig. 4). No metaphases containing other than human chromosome

FIG. 1.—Light microscopy of hematoxylin-stained fixed cultured cells from the 50th passage. Overlapping nuclei and several mitotic figures are found in the dense areas of the culture. × 400.

FIG. 2.—Phase-contrast microscopy of a multinucleate giant cell. × 340.

FIG. 3.—Zymogram of glucose-6-phosphate dehydrogenase on cellulose acetate gel (Cellogel). Channels—1: A9 mouse cell lines; 2: HeLa cells grown in monolayer; 3: HeLa cells in suspension culture; 4: HEEM cells; 5: primary skin fibroblasts derived from a man with G6PD B+ phenotype.

banding patterns were observed. In more than 90% of the metaphases examined, Y chromosomes were present.

Occasionally, in metaphases containing large numbers of chromosomes 2 or 3 Y chromosomes could be visualized.

*Growth characteristics*

Population doubling time of the viable cells in the culture was estimated as 2 days. Numerous mitoses could be found in areas of high cell density, suggesting a lack of contact inhibition.

Cell densities of up to $4 \times 10^5$ cells/cm$^2$ of glass surface were found. The cells grew easily in medium containing 1% FCS and they appeared to grow only slightly slower than the cells maintained in 10% FCS. Absolute plating efficiency of the cells ranged from 80 to 90%. Single cells seeded in soft agar grew out to macroscopically visible colonies (Fig. 5).

*Tests for tumorigenicity*

The X-irradiated hamsters started to die after 2 weeks. After 4 weeks, the surviving animals were in poor health and had to be sacrificed. On autopsy no tumours were found. Some of the non-irradiated animals were followed for 5 months without evidence of developing tumours. Neither the homozygous nor the heterozygous nude mice developed tumours in 5 months. Out of each group, one i.p.-injected and one s.c.-injected animal was sacrificed. Macroscopically no tumours were found.

*Virological studies*

Because it was known from a previous publication (Zurcher *et al.*, 1975) that the HEEM cells contain antigens cross-reacting with antibody against the major polypeptide of the simian sarcoma virus, we looked for further evidence of the presence of oncornavirus in these cells. These viruses have been shown to be involved in tumorigenesis in various vertebrates, especially in rodents and in poultry. Oncornaviruses contain 70S

FIG. 4.—Metaphase with 70 chromosomes with a human banding pattern. Some of the chromosomes have clearly undergone structural rearrangements.

FIG. 5.—Colony obtained 14 days after seeding single HEEM cells in soft agar. ×34.

FIG. 6.—The activity of reverse transcriptase along a sucrose gradient. Panels—A: 0·45 g of HEEM cells was processed to yield a membrane fraction that was subsequently centrifuged to equilibrium; B: the membrane fraction from 0·45 g of cells was treated with the detergent NP40 before equilibrium centrifugation. ■: Incorporation of [$^{3}$H]TTP into TCA-precipitable material; ●, density of the gradient fractions.

FIG. 7.—Simultaneous detection test on the core material from 5 g of HEEM cells. The newly synthesized DNA was sedimented in a sucrose gradient either before (○) or after (●) treatment with RNase. Arrows indicate the positions in a parallel gradient of 70S RNA from the B77 strain of RSV and 28S ribosomal RNA respectively.

FIG. 8.—Electron micrograph of a HEEM cell. ×3000.

RNA and an enzyme capable of synthesizing DNA from an RNA template.

(*a*) *Biochemical assays*.—The HEEM cells appeared to contain an enzyme capable of utilizing oligo (dT)-poly(rA) for synthesis of DNA.

As can be seen from Fig. 6A, the activity of the enzyme was associated with material banding in a sucrose gradient at a density of 1·16. After treatment with the non-ionic detergent Nonidet P-40 the enzyme activity banded at a density of 1·23 (Fig. 6B). From these results it seemed possible that the enzyme was associated with an oncornavirus-like particle.

We therefore performed a simultaneous detection assay to see whether the enzyme was associated with 70S RNA. As can be seen from Fig. 7, part of the newly synthesized DNA co-sedimented with an RNase-sensitive molecule at 70S.

(*b*) *Electron microscopy*.—When examined with the electron microscope, thin sections of the cultured cells showed that cytoplasmic organelles such as mitochondria and endoplasmic reticulum were scant (Fig. 8). In spite of an extensive search for " budding " virus, no particles were found in hundreds of cell-sections.

In the supernatant tissue culture medium, particles were occasionally found in the thin sections. These particles, occurring in the fraction with a density of 1·16, had a size (100 nm) and morphology resembling those of oncornavirus (Fig. 9).

### DISCUSSION

This paper deals with a rapidly growing cell line derived from a human

FIG. 9.—Electron micrographs of virus-like particles in the 1·16 density fraction of the culture medium. Bar represents 100 nm.

chondrosarcoma. The cell line is apparently uncontaminated with HeLa cells. Although many types of human sarcoma cells have been described (Aaronson *et al.*, 1970; Giraldo *et al.*, 1971; Ioachim, 1970; McAllister *et al.*, 1971; McAllister *et al.*, 1975; Morton *et al.*, 1969; Ponten and Saksela, 1967; Rasheed *et al.*, 1974; Stewart *et al.*, 1972; Winters *et al.*, 1974), the establishment of continuous cell lines from human skeletal tumours has only been reported infrequently (Giraldo *et al.*, 1971; McAllister *et al.*, 1971; Ponten and Saksela, 1967; Winters *et al.*, 1974). In the cell line reported here, the cells had an epithelioid appearance and, when the cultures attained confluency, a number of giant cells with up to 10 nuclei were occasionally found. The cells had a high plating efficiency of 80–90%. They were aneuploid, with chromosome numbers ranging from 63 to 85. This aneuploidy is probably not the result of the prolonged exposure of the cells to culture conditions, since it was apparent in the 9th passage when the first karyotypic examination was made. It appeared that the cultures could grow to high densities (up to $4 \times 10^5$ viable cells/cm$^2$) and that the cells apparently lacked contact inhibition (Fig. 1). These observations, and the growth potential in low serum concentrations and in soft agar, indicate that the cells have a number of properties of transformed cells.

The cultured cells failed to grow when inoculated intracutaneously in the cheek pouch of either irradiated or non-irradiated hamsters, or after s.c. or i.p. injection in either homo- or heterozygous nude mice.

The cell line described in the present report was included in a study on the presence of oncornaviral antigens in human tumour cells (Zurcher *et al.*, 1975). In Fig. 1 of that paper, the cells of the HEEM cell line were shown to react with an antiserum against the major polypeptide of the simian sarcoma virus, and to a lesser extent with antiserum to Rauscher murine leukaemia virus. These antigens were present immediately after starting the culture, and they are still demonstrable about 50 passages later.

In tissue cultures there is always the possibility that a virus is introduced into the cells, for example by bovine trypsin or serum. From the previous publication (Zurcher *et al.*, 1975), it seems improbable that infection with bovine leucosis virus has occurred. Also, it appears that the viral antigens present in the HEEM cells are more closely related to simian sarcoma viral proteins than to those of the Rauscher virus. No simian sarcoma virus has been present in the laboratory in which the HEEM cells were cultured.

The biochemical experiments reported in the present paper indicate that the HEEM cells contain a low number of particles with properties of oncornavirus (particles with a density of 1·16 with "cores" of density 1·23 associated with a reverse-transcriptase-like enzyme and with 70S RNA). However, in thin-section electron microscopy of the cultured cells, "budding" viral particles could not be found. Occasionally the presence of oncornavirus-like particles was demonstrated by means of thin-section electron microscopy of material derived from the culture medium.

The authors wish to thank Mrs N. Koopman-Broekhuyzen and Mr F. Prins for technical assistance, and Miss L. M. M. Wijnen of the Department for Anthropogenetics, University of Leiden, for assistance in the study of the isoenzyme patterns. The cytogenetic studies were done in conjunction with Dr P. Pearson of the Department for Anthropogenetics, University of Leiden. The biotechnical work was done by Mr F. Leupe of the Department of Medical Microbiology, University of Leiden. We wish to thank Mr W. Beens for technical assistance in electron microscopy and Mrs M. W. J. Broekhuizen-Dubbelaar and Mrs E. M. de Groot-van der Hoeven for the preparation of the manuscript.

This research was supported by the Queen Wilhelmina Foundation against Cancer.

### REFERENCES

AARONSON, S. A., TODARO, G. J. & FREEMAN, A. E. (1970) Human Sarcoma Cells in Culture. *Exp. Cell Res.*, **61**, 1.

GARTLER, S. M. (1968) Apparent HeLa Cell Contamination of Human Heteroploid Cell Lines. *Nature, Lond.*, **217**, 750.

GIRALDO, G., BETH, E., HIRSHAUT, Y., AOKI, T., OLD, L. J., BOYSE, E. A. & CHOPRA, H. C. (1971) Human Sarcomas in Culture. *J. exp. Med.*, **133**, 454.

IOACHIM, J. L. (1970) Tissue Culture of Human Tumors: its Use and Prospects. *Path. Ann.*, **5**, 217.

KARNOVSKY, M. (1961) Simple Methods for Staining with Lead and High pH in Electron Microscopy. *J. biophys. biochem. Cytol.*, **11**, 729.

LEVINE, E. M. (1974) Mycoplasma Contamination of Animal Cell Cultures: a Simple, Rapid Detection Method. *Exp. Cell Res.*, **74**, 99.

LUFT, J. (1961) Improvements in Epoxy Resin Embedding Method. *J. biophys. biochem. Cytol.*, **9**, 400.

MCALLISTER, R. M., GARDNER, M. B., GREENE, A. E., BRADT, C., NICHOLS, W. W. & LANDING, B. H. (1971) Cultivation *in vitro* of Cells Derived from a Human Osteosarcoma. *Cancer, N.Y.*, **27**, 397.

MCALLISTER, R. M., NELSON-REES, W. A., PEER, M., LAUG, W. E., ISAAKS, H., GILDEN, R. V., RONGEY, R. W. & GARDNER, M. B. (1975) Childhood Sarcomas and Lymphomas. Characterization of New Cell Lines and Search for Type-C virus. *Cancer, N.Y.*, **36**, 1804.

MEERA KHAN, P. (1971) Enzyme Electrophoresis on Cellulose Acetate Gel: Zymogram Patterns in Man–Mouse and Man–Chinese Hamster Somatic Cell Hybrids. *Archs Biochem. Biophys.*, **145**, 470.

MILLER, M. F., ALLEN, P. T. & DMOCHOWSKI, L. (1973) Quantitative Studies on Oncornaviruses in Thin Sections. *J. Gen. Virol.*, **21**, 5768.

MORTON, D. L., HALL, W. T. & MALMGREN, R. A. (1969) Human Liposarcomas: Tissue Culture Containing Foci of Transformed Cells with Viral Particles. *Science, N.Y.*, **165**, 813.

VAN DER PLOEG, M. & PLOEM, J. S. (1973) Filter Combinations and Light Sources for Fluorescent Microscopy of Quinacrine Mustard and Quinacrine Stained Chromosomes. *Histochemie*, **33**, 61.

PONTEN, J. & SAKSELA, E. (1967) Two Established *In vitro* Cell Lines from Mesenchymal Tumors. *Int. J. Cancer*, **2**, 434.

RASHEED, S., NELSON-REES, W. A., TOTH, E. M., ARNSTEIN, P. & GARDNER, M. B. (1974) Characterization of a Newly Derived Human Sarcoma Cell Line (HF-1080). *Cancer, N.Y.*, **33**, 1027.

SCHLOM, J. & SPIEGELMAN, S. (1971) Simultaneous Detection of Reverse Transcriptase and High Molecular Weight RNA Unique to Oncogenic RNA Viruses. *Science, N.Y.*, **174**, 840.

STEWART, S. E., KASNIC, G., DRAYCOTT, C. & BEN, T. (1972*a*) Activation of Viruses in Human Tumors by 5-Iododeoxyuridine and Dimethyl Sulfoxide. *Science, N.Y.*, **175**, 198.

STEWART, S. E., KASNIC, G., DRAYCOTT, C., FELLER, W., GOLDEN, A., MITCHELL, E. & BEN, T. (1972*b*) Activation *In vitro* by 5-Iododeoxyuridine of a Latent Virus Resembling C-type Virus. *J. natn. Cancer Inst.*, **48**, 273.

WINTERS, W. D., NERI, A. & MORTON, D. L. (1974) New Cell Line Derived from a Human Chondrosarcoma. *In Vitro*, **10**, 70.

WITKIN, S. S., OHNO, T. & SPIEGELMAN, S. (1975) Purification of RNA-instructed DNA Polymerase from Human Leukemic Spleens. *Proc. natn. Acad. Sci. U.S.A.*, **72**, 4133.

ZURCHER, C., BRINKHOF, J., BENTVELZEN, P. & DE MAN, J. C. H. (1975) C-type Virus Antigens Detected by Immunofluorescence in Human Bone Tumour Cultures. *Nature, Lond.*, **254**, 457.